# Perspectives of family physicians towards access to lung cancer screening for individuals living with low income – a qualitative study

**DOI:** 10.1186/s12875-020-01354-z

**Published:** 2021-01-07

**Authors:** Ambreen Sayani, Mandana Vahabi, Mary Ann O’Brien, Geoffrey Liu, Stephen W. Hwang, Peter Selby, Erika Nicholson, Aisha Lofters

**Affiliations:** 1 MAP Centre for Urban Health Solutions, Li Ka Shing Knowledge Institute, St. Michael’s Hospital, Toronto, Ontario Canada; 2 Women’s College Research Institute, Women’s College Hospital, Toronto, Ontario Canada; 3 Daphne Cockwell School of Nursing, Ryerson University, Toronto, Ontario Canada; 4 ICES, Toronto, Ontario Canada; 5 Department of Family and Community Medicine, University of Toronto, Toronto, Ontario Canada; 6 Dalla Lana School of Public Health, Toronto, Ontario Canada; 7 Princess Margaret Cancer Centre, Toronto, Ontario Canada; 8 Department of Medicine, University of Toronto, Toronto, Ontario Canada; 9 Campbell Family Research Institute, Centre for Addiction and Mental Health, Toronto, Ontario Canada; 10 Department of Psychiatry, University of Toronto, Toronto, Ontario Canada; 11 Canadian Partnership Against Cancer, Toronto, Ontario Canada; 12 Department of Family Medicine, Women’s College Hospital, Toronto, Ontario Canada

**Keywords:** Lung-cancer screening, Health equity, Family physician perspectives

## Abstract

**Background:**

Individuals living with low income are less likely to participate in lung cancer screening (LCS) with low-dose computed tomography. Family physicians (FPs) are typically responsible for referring eligible patients to LCS; therefore, we sought to understand their perspectives on access to lung cancer screening for individuals living with low income in order to improve equity in access to LCS.

**Methods:**

A theory-informed thematic analysis was conducted using data collected from 11 semi-structured interviews with FPs recruited from three primary care sites in downtown Toronto. Data was coded using the Systems Model of Clinical Preventative Care as a framework and interpretation was guided by the synergies of oppression analytical lens.

**Results:**

Four overarching themes describe FP perspectives on access to LCS for individuals living with low income: the degree of social disadvantage that influences lung cancer risk and opportunities to access care; the clinical encounter, where there is often a mismatch between the complex health needs of low income individuals and structure of health care appointments; the need for equity-oriented health care, illustrated by the neglect of structural origins of health risk and the benefits of a trauma-informed approach; and finally, the multiprong strategies that will be needed in order to improve equity in health outcomes.

**Conclusion:**

An equity-oriented and interdisciplinary team based approach to care will be needed in order to improve access to LCS, and attention must be given to the upstream determinants of lung cancer in order to reduce lung cancer risk.

**Supplementary Information:**

The online version contains supplementary material available at 10.1186/s12875-020-01354-z.

## Background

It is estimated that one in 15 Canadians will be diagnosed with lung cancer over their lifetime, making it one of the most frequently diagnosed cancers amongst Canadians [1]. Lung cancers are usually detected at an advanced stage (stage III or stage IV) when the chances for curative therapy are low. Consequently, lung cancer contributes to a quarter of all cancer -related deaths in Canada [1]. Lung cancers that are diagnosed at an early stage require less invasive therapy, are more amenable to treatment, and associated with lower mortality [2]. The National Lung Screening Trial (NLST) published in 2011 demonstrated that screening with low-dose CT (LDCT) versus x-ray led to detection of more lung cancers and fewer lung cancer deaths after seven years of follow-up. In 2020, The Dutch–Belgian lung-cancer screening trial (Nederlands–Leuvens Longkanker Screenings Onderzoek [NELSON]), demonstrated a reduction in lung cancer specific mortality for individuals who underwent lung cancer screening at eleven years of follow up [3]. In Canada, it is currently a health system priority to plan and implement lung cancer screening (LCS) for individuals at a high-risk of developing lung cancer, i.e. individuals between the ages of 55 and 74 years who have a 30 pack year smoking history (pack-year defined as the [average number of cigarette packs smoked daily] x [number of years smoking]) or quit smoking less than fifteen years ago [4]. In Ontario, this mandate is currently undergoing pilot testing by the provincial healthcare agency Ontario Health across four locations in the region.

Smoking remains the single largest risk factor for developing lung cancer. Importantly, smoking is more prevalent amongst individuals living with low income [5] who by extension are more likely to have lung cancer [6]. In Canada, evidence points to the unequal utilization of cancer screening programs by individuals living with low income [7]. Inequities in cancer screening arise due to a lack of awareness of cancer screening programs in socially disadvantaged population groups and resources required to access care such as, transportation and childcare [7]. Importantly, however, these differences reflect an unequal distribution of the social determinants of health (SDH) such as income, and the unfair distribution of resources that shape opportunities to access and utilize cancer care [8, 9]. Much like inequities in other cancer screening programs (breast, colon, cervical) [10] participation in LCS is influenced by conditions of social disadvantage, such that individuals living with low income are less likely to use the preventative test [11].

Referrals to all types of cancer screening are typically make by family physicians (FPs) who are situated between individuals at-need of receiving cancer screening on one hand, and access to cancer screening through the cancer care system on the other hand. FPs can both facilitate access to cancer screening or serve as a barrier [12], and evidence points to increase utilization of cancer screening based on a positive relationship with FPs, adequate time in the clinical encounter to make recommendations towards preventative care and the presence of automated reminders in the patients electronic medical record (EMR) [13–15]. Given the disproportionate burden of lung cancer in populations living with social disadvantage, it is important to understand the perspectives of physicians on access to LCS for individuals living with low income. This knowledge can provide a window into the complexity of tailoring preventative care to populations experiencing both social and health inequities and therefore can inform the delivery of equitable cancer screening to high-risk populations.

## Methods

### Study design

Research Ethics Board approval for the study was obtained from Unity Health Toronto. We used theoretical thematic analysis [16] as a methodology in order to conceptualize, collect, organise and interpret data using synergies of oppression theoretical lens [17] and guided by the Systems Model of Clinical Preventive Care by Walsh and McPhee [18]. The synergies of oppression is an approach to conceptualizing the intersection of social identities (gender, race, disability etc.) with the SDH (income, education etc.) and social geography (service accessibility, rural/urban location) [17]. As a conceptual tool, this lens allows us to envision income as one of many intersecting identities that influence social location and ultimately the opportunities to access and utilize care. The Systems Model of Clinical Preventive Care [18] is a framework that focuses on the physician-patient interaction, and the factors that inhibit or promote the completion of preventative health care activity at the patient, physician and system level.

### Participant recruitment and setting

We used a purposive sampling technique and invited 80 FP’s practising across three primary care settings in downtown Toronto that serve a wide catchment of patients. Each potential participant was contacted by the last author (AL) through an e-mail which included information about the study, and invited participants to an interview to share their perspectives on the barriers and facilitators to LCS for individuals living with low income. AL is a FP and clinical epidemiologist. All eligible participants were contacted (by AL) through e-mail three times between June 2018 to July 2019. FPs who agreed to participate provided verbal consent and were interviewed by the principal researcher and first author (AS). Apart from one participant, no prior association between the principal researcher and participants existed prior to study commencement. AS is a female medical doctor and critical qualitative researcher. Both AS and AL research health inequities. A total of 11 FP’s (three males, eight females) were recruited. FPs described their individual practice to consist of a high prevalence of those living with low income (S1), experiencing homelessness (S2) and/or suffering from multiple addictions and psychosocial problems (S3). Table 1 shows participants’ gender, practice setting and self-reported percentage of practice population experiencing low income. We did not collect further demographic data from participants.

**Table 1:** Participant Characteristics

Participant code	Sex	Practice site	Percentage of practice experiencing low income
FP1	F	S3	> 75%
FP2	F	S1	< 25%
FP3	F	S1	< 25%
FP4	F	S1	25–50%
FP5	F	S1	< 25%
FP6	M	S1	50–75%
FP7	F	S1	< 25%
FP8	F	S1	> 75%
FP9	F	S1	50–75%
FP10	M	S1/S2	> 75%
FP11	M	S1	< 25–50%

### Data collection

Data were collected through semi-structured interviews which were guided by the conceptual framework of the study. The interview guide was developed by two researchers (AS, AL) and reviewed by a third member of the team (MAO). MAO is a qualitative researcher with expertise in knowledge translation. All interviews were conducted over the telephone by the first author (AS) and lasted between 30 to 45 min. The interview guide was pilot-tested on one FP and some questions were rephrased for clarity. During the interviews, participants were asked about their practice setting, approach to cancer screening in general, and their perspectives on LCS. Specifically, this included questions about lung cancer risk, barriers to care for individuals living with low income, and experiences referring patients to LCS. In addition, all FPs were asked about ways in which access to LCS can be made more equitable. Interviews were audio-recorded with participants’ permission and transcribed by a professional transcriptionist. Transcripts were reread for accuracy by comparing to field notes (made by AS during the interviews) and were subsequently entered into a qualitative software program (NVivo version 12) for data management. All participants received a $25 gift card as honorarium.

### Data analysis

**Fig. 1:**
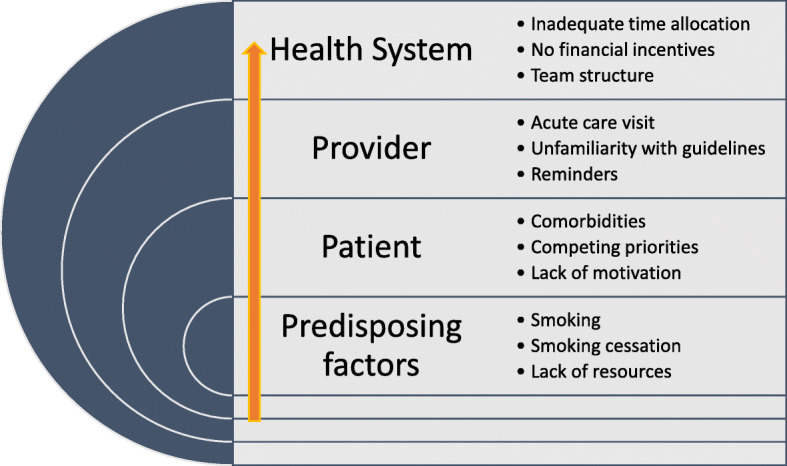
Coding tree based on the Systems Model of Clinical Preventative Care and sample codes

We followed the six steps of thematic analysis as outlined by Braun and Clarke [16] and followed the trustworthiness criteria outlined by Nowell et al. [19]. Transcripts were read and reread (by AS, AL) before coding began. An overarching coding framework was manually created in NVivo 12 corresponding to the Systems Model of Clinical Preventative Care [18]. Two researchers (AS, AL) independently identified initial codes by reading all transcripts. Codes were then compared, discussed and modified before applying line-by-line coding to the texts (AS). Additional codes were developed and modified (AS, AL) as the coding progressed until all lines of text had been completely coded. Informational saturation was reached with the data [20]. Figure 1 shows the structure of the coding tree with sample codes. Regular teleconference and in-person team meetings provided opportunities to peer debrief about the developing themes [21]. The multidisciplinary nature of the research team created opportunities to discuss differences in interpretation and enhanced reflexivity. Using thematic analysis as a methodology allowed us to capture any outlier themes. Specifically, we identify equity-oriented healthcare (EOHC) as an outlier theme noted by the infrequency with which it was addressed. We discuss this theme in more detail below. The final themes were reached through iterative cycles between four authors (AS, AL, MAO, MV) and validated by the larger research team. MV is registered nurse and social epidemiologist. The extended research team comprised of clinical oncologists (GL), clinician scientists in addiction and mental health (PS) and homelessness (SH) as well a federal level policy maker that is involved in the implementation of LCS in Canada (EN).

## Results

**Fig. 2:**
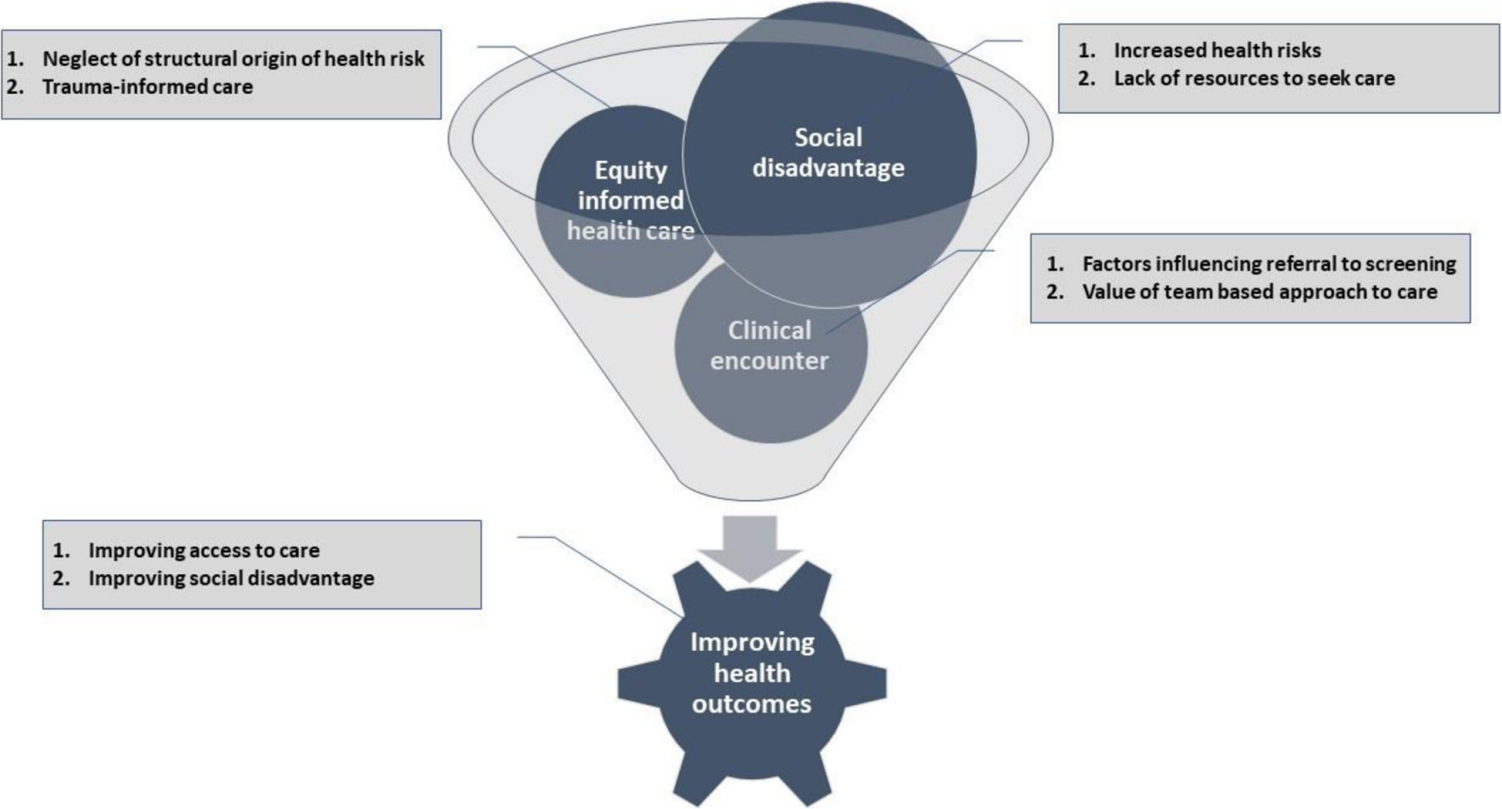
Themes and Subthemes of the Study

Figure 2, shows a thematic map connecting our four main themes: Social disadvantage, the clinical encounter, equity-oriented health care, and improving health outcomes.

An overview of the themes is discussed below. The complete set of themes, subthemes and illustrative quotes that were derived from the transcripts is shown in Table 2.

**Table 2:** Themes, Subthemes, and Illustrative Quotes from Interview Transcripts

Theme - subtheme	Illustrative quotes
Social disadvantage:	
1. Increased health risks.	“I think my practice reflects what we know in the general population which is that as you go down the income scale and down the scale of housing security and other forms of social security that type of risky behaviour tends to increase. But I think that is absolutely true in my practice as well. So (individuals with) major mental health diagnoses are much more likely to smoke, people who are living in poverty who experienced homelessness, people who have experienced trauma, people who experience other forms of social pressure like racism, people who are racialized, people who…you know identify as LGBT or trans. I mean I’d say people from any one of these groups have a higher likelihood of smoking.” (FP10)
2. Lack of resources to seek care.	“They’re all doing work hours, so people who work shiftwork, people who are taking care of children, all of these sorts of folks who are generally going to be less socially advantaged. These people are not able to take off time because they’re not necessarily protected in their jobs or have job security. These folks are not going to come to my office with all this information saying I want a CT for lung cancer screening.” (FP11) “For socially vulnerable patients (…)I need to be more attune to them in terms of how they can be notified of their appointments so they’ll have phones that sometimes are connected, sometimes aren’t. Some of them much prefer to have appointment notifications through email because they can. They have their computers and they can check email at like the shelters they’re staying at, whereas for others, that won’t work for them at all. So it’s hard because there’s not like one answer that works best for this particular population.” (FP2)
Clinical encounter:	
1. Factors influencing referral to screening.	“They only seek medical care episodically in crisis that they’re often challenging to accept into family practices because of time restrictions. Our model of seeing patients isn’t very amendable to super complex patients who have health complex needs as well as socio-economic needs so it’s hard to build a relationship. It’s hard to keep appointments where you need to be at an appointment at a certain time.” (FP5) “I mean our appointments are 15 min usually. And I mean we try to practice patient-centered care. So for instance, I had somebody who had a major medical issue and I’m quite focused on managing that. Making sure that she doesn’t go into liver failure, but her priority coming in is to deal with housing issues, and so those are two large topics and therefore like bringing up that she’s due for a screening test there’s not always time and I’m distracted by these other big issues that I think are potentially more urgent. So this distracts my attention.” (FP8)
2. Value of team based approach to care.	“If there was maybe some type of patient navigator who is quite familiar with the system and was a bit of a quarterback to coordinate the appointments and help people with transit and putting the pieces together and identifying the gaps to bring to the social worker or the teams that might help.” (FP4) “At a Community Health Centre they have social workers and like they are part of a team. So if you’re in a primary care model where you’re part of a team it is possible to help a patient get a little bit more stabilized, so if it’s a mental health issue than you know have them either see a psychiatrist If it’s social situation then yeah, I wouldn’t manage that myself but I would refer to the social worker and we have social workers.” (FP1) “Maybe hooking them up with a social worker or a counsellor …(and) maybe having a coordinator involved just to make sure they make their appointments.” (FP7)
Equity-oriented health care:	
1. Neglect of structural origin of health risks.	“I really try to help people understand what an addiction truly is. I’m like this is not a judgment on you that you’re still smoking. It’s not because you’re lazy or just don’t have enough willpower, like when you started smoking that early in your life you know it really changed your brain.” (FP3) “I think probably a combination of boredom, of culture, of you know, it (smoking) is something that’s done in peer groups so I think that it’s more socially acceptable.”(FP5) “Factory workers, people who work in a warehouse, in most of these kinds of jobs it is just the acceptable way to take a break… Some people are smoking because they need to stay awake because they have a very long-distance drive … They don’t usually go anywhere (with smoking cessation) until they change their job.” (FP9)
2. Trauma- informed care.	“I’d say that in our first few encounters I noted the smoking history. I gently flagged it as an issue but I spent very little time on it in our first few encounters. Well really I mean first two years of knowing him because we were really working on stabilizing all the other things going on in his life and those were clearly his priorities and clearly my priorities; right. I didn’t think the smoking mattered all that much in the context of everything else that was going on for him which was far greater threats to his health than the smoking. Progressively over time his social situation really stabilized in very significant ways. He became housed, he got on ODSP so he got steady income…the conversations around smoking just started to just take up more time in our interactions progressively and you can probably almost put a like linear graph to that in terms of how much time they were taking up and you could just sort of see smoking rising up as an issue in terms of the list of priorities as other things kind of stabilized.” (FP10)
Improving health outcomes:	
1. Improving access to care.	“Taxi chits … phone call reminders, you know because often we get patients who will miss my appointments.” (FP8) “Different language literature, pictorial designs that makes it easy to understand. Maybe a navigator in different languages. Community programs. Community websites.” (FP9) “I think automating it where possible so that the smoking history, anyone with a heavy smoking history will be automatically flagged.” (FP6) “Compensation for physicians when we make phone calls or do emails cause it’s not just outside of our work time like it cuts into our work-life balance, but we do it, but I think we’d do a little bit more of it maybe if there was some sort of acknowledgement.”(FP8)
2. Improving social disadvantage.	“How likely are they (low income patients) to tolerate chemotherapy or treatment or surgery? You know going through those very, very intensive treatments or follow ups; and it’s not easy when they’re coming home to a shelter bed or they’re coming home and they’re choosing. A lot of the time they have to make choices between food and bills and those sort of costs are very, very significant. So, I think one question is are we screening them enough? And the other question is are we supporting them enough to get treatment? So, even if I had, if I was able to get everyone screened at the point of detecting lung cancer what then would happen to these patients?” (FP11) “Help people get housed and help them get a better income quite honestly. I mean I don’t think the answer lies in tweaks in a screening program. I think that these are systemic issues and I think it would be a mistake, and I think it would reinforce the systemic problems that we have to sort of say you know, we can finance this population in how we offer this program….(and create) a diversion of resources to this type of screening (and away from) what’s most important for building a foundation for good health.” (FP10)

### Social disadvantage

FPs associated conditions of social disadvantage with: (i) increased health risks, and (ii) lack of resources required to access health services.

When speaking of health risks, FPs described the clustering of social disadvantage in populations that were more likely to smoke. Specifically, FPs recounted how a poor quality of distribution of the SDH such as income and housing, and marginalized social identities such as race, and sexual orientation were specifically linked with risky lifestyle behaviours and a higher incidence of smoking.

FPs also described the variety of resources that are needed to access health services. Specifically, FPs mentioned the need for flexible working hours and access to childcare as a prerequisite to seeking preventative health care. Additionally, FPs spoke of the difficulty in communicating health services to patients living with low income given their lack of a consistent mailing address, inability to have and access emails, and a high frequency of disconnected phone lines.

### Clinical encounter

FP’s described clinical encounters with low income patients as challenging. Specifically, FPs discussed: (i) factors influencing their ability to refer patients to screening, and (ii) the value of a team based approach to care.

FPs mentioned that care for socially disadvantaged patients was frequently episodic and centred around crisis management. When speaking of the clinical encounter, FPs described how the structure of clinic appointments such as, a fifteen minute time slot created a mismatch to the often complex and underlying health needs of patients who came from a low income demographic. Subsequently, the focus of the short clinic encounter was frequently left to managing acute health needs rather than preventative health care.

Several FPs considered that shared clinical management, particularly with other front line care providers could alleviate some of the pressures of delivering holistic care within a short clinic appointment. Specifically, FPs described the role of nurses and social workers in facilitating appointments, transportation and cancer screening.

### Equity-oriented health care (EOHC)

Equity-oriented healthcare (EOHC) s an approach to improving health equity at the point of care by creating: “safe and respectful environments while tailoring health care to fit the needs, priorities, history, and contexts of individual patients and populations served” [22]. At the patient-provider interaction level, EOHC is responsive to social structural inequalities which underpin health differences by using a trauma-informed approach to the delivery of care [22]. In our analysis, we found that FPs approach to managing health risks, such as smoking behaviour fell into one of the following categories: (i) a neglect of the structural origin of health risks, or (ii) trauma-informed care.

While all FPs linked conditions of social disadvantage with a higher incidence of smoking, several FPs described smoking as a personal choice that was influenced by peers and working conditions. For these FPs, the locus of healthy behaviour resided within the individual and smoking was a choice that patients could control or influence, thereby neglecting the underlying societal structures that shape health risk or the biological basis of addiction which has evidence based treatments covered by Ontario Drug Benefits.

One FP described their approach to care as being centred around the living conditions and social needs of patients, with a focus on empowering patients by enhancing their SDH. For this FP, secure housing and stable income were pre-conditions to smoking cessation. This description of care falls within the realm of trauma-informed care through theacknowledgement and address of the effects of structural violence on individuals health and health seeking choices [22].

### Improving health outcomes

All FPs discussed barriers to LCS and described ways to enhance health outcomes through LCS. These perspectives towards health promotion fell into one of two groups: (i) improving access to care, or (ii) improving social disadvantage.

When discussing potential interventions to address barriers to LCS, most FP’s suggested ways in which to increase access to care such as providing taxi fare or phone reminders. FPs also discussed enhancing communication about the program through community outreach and multilingual information resources. Further, FPs described ways in which they could be facilitated to refer patients to LCS through electronic medical record (EMR) reminders and financial incentives to care for complex patients.

Two FPs were less concerned with improving access at the point of care. Rather, one FP spoke of his concern about screening low income patients for lung cancer given that a lack of appropriate housing, or secure income could undermine the ability of patients to follow through on therapy for lung cancer if indeed the screening result came out positive. Another FP questioned the allocation of resources towards screening all together, arguing that resources would be better spent on housing and income security in order to create a better foundation for overall health.

## Discussion

Historically, individuals with lower levels of income are less likely to participate in preventative health checks such as cancer screening [10]. For LCS this is particularly problematic since the highest incidence of lung cancer is found in populations disadvantaged by a variety of SDH such as income and education. LCS therefore presents two very specific challenges: (i) the highest prevalence of disease among those with the greatest degree of social disadvantage; and (ii) the lowest uptake of screening in the highest risk target population.

Our work highlights barriers to screening from the physician’ perspective such as, the nature of acute care visits, multiple patient comorbidities, and a lack of reminders through EMRs. None of these findings are novel, and have been previously documented in studies which have described physician barriers and facilitators to screening for other cancers such as breast [13], cervix [14] and colon [15]. In our analysis however, we apply the synergies of oppression theoretical framework in orderto illuminate how the need and ability to access care is shaped by intersecting oppressions resulting in social disadvantage. We describe therefore the need for a reorientation of services and resource allocation in order to achieve health equity. This is one of the strengths of using a theoretical thematic analysis as it is able to produce evidence that is relevant to the development of policy by illuminating experiences and perspectives in the real-world setting of patients, providers and policy makers [23]. Our qualitative study which contextualizes the lived experiences of poverty and the choice to participate in LCS for individuals living with low income in Ontario is described elsewhere [24].

Although our research question was focused on understanding physicians’ perspectives towards LCS for patients living with low income, FPs described how other intersecting identities (such as, race and sexual orientation) and SDH (such as, education and housing) were often associated with low income and higher rates of smoking. The frequent presence of multiple comorbidities represented a further challenge to care, particularly in the context of a mismatch between time allocated to provide care, and the complex care needs of socially disadvantaged patients. FPs frequently emphasized the need for a more integrated approach to care that included a larger interprofessional team of nurses, social workers, and patient navigators as one way to improve screening rates. FPs also described the need for resources such as transportation and communication tools as ways to improve access to screening, and subsequently health outcomes. However, few FPs questioned the underlying structural processes such as, jurisdictional policies that influence the unequal distribution of income. These policies include affordable housing, a living wage and better support for individuals facing discrimination and societal exclusion. No FPs spoke of the role of the tobacco industry and contraband manufacturers and the structural violence of large companies that profit from addicting the population for profit [25].

There are several implications of our findings. First, socially disadvantaged populations cannot be accommodated by a one size fits all approach to health care delivery. Our findings suggest that interprofessional teams of health providers, such as is found in community health centres (CHCs) where providers work on a salaried basis rather than fee-for-service may be ideally positioned to provide wraparound health and social services. This integrated approach has been shown to improve cancer screening [26] by empowering patients to enhance their overall opportunities to attain good health. Whether this model will work to improve uptake of LCS has yet to be tested. Second, for populations facing multiple oppressions, EOHC is an important way to improve health equity at the point of care [22]. By understanding that health behaviour is shaped more by the unequal distribution of the SDH through policies that promote inequities and social discrimination, rather than personal choice and control over life chances, providers will be able to avoid perpetuating cycles of poverty and oppression. EOHC thus forms an important way of delivering culturally safe, and contextually tailored care, through a trauma-informed approach [22]. This has been shown to improve care experiences for socially disadvantaged groups towards a goal of health equity [22]. Finally, improving health outcomes through LCS will require a multiprong approach that will require a mix of interventions which improve access to care, as well as an emphasis on upstream policies which will enhance the quality and distribution of the SDH. This underscores the need to transform healthcare to include the lived experiences of those who are marginalized and often excluded from the design of health interventions in order to improve the offer of cancer screening and appropriate uptake as well as a reduction in cancer risk.

### Strengths and limitations

We used a theoretical thematic analysis as a way of analysing our data within a social context [16]. There are several strengths to this approach, which we demonstrated in our study as the ability to analyse physician perspectives in the social context of lung cancer risk. The pivot of this analysis then is not limited to improving access to LCS, but rather we illuminated the need to focus simultaneously on both upstream and midstream factors in order to understand and respond to the social determinants of lung cancer risk. We are limited by a small sample size of FPs practising in primary care settings in a single city. As a qualitative study, we do not expect our work to be generalizable, however we believe that by describing our study setting and participant population, our findings will be transferable to other primary care locations. As a next step, and as pilot-testing of LCS expands across the province of Ontario, it will be important to understand if FP demographics, and racial/ethnic identity influence perspectives on access to LCS for individuals living with low income and how these perspectives vary based on the sociodemographic profile of the geographic area which they serve.

## Conclusion

A team-based approach to clinical care that addresses the social determinants of health and facilitates overall health and wellbeing can form the basis of an equity-informed way to enhance access to LCS. All health care providers must recognise that the living and working conditions which predispose patients to ill-health and influence their ability to access care are shaped by jurisdictional policies, and therefore must be acknowledged in the clinical encounter and addressed at the political level.

## Supplementary Information

**Additional file 1.**

**Additional file 2.**

## Data Availability

The datasets used and/or analysed during the current study are available from the corresponding author on reasonable request.

## Abbreviations

LCS: Lung cancer screening
FP: Family physician
LDCT: Low-dose CT
NLST: National Lung Screening Trial
NELSON: Dutch–Belgian lung-cancer screening trial (Nederlands–Leuvens Longkanker Screenings Onderzoek
SDH: Social determinants of health
EOHC: Equity-oriented healthcare
EMR: Electronic medical record
