# Supplementary material for: Perspectives of family physicians towards access to lung cancer screening for individuals living with low income – a qualitative study
Source: BMC Fam Pract. 2021 Jan 7;22:10. doi: 10.1186/s12875-020-01354-z (PMC7791696; doi:10.1186/s12875-020-01354-z)
Supplement: Supplementary file 1 — Additional file 1. [file 12875_2020_1354_MOESM1_ESM.docx]

**Supplemental File S1: Interview Guide - Providers**

In this interview, we will be discussing using low-dose CT for lung cancer screening.

[Obtain verbal consent on audiotape for participation in interview]

1. What has been your experience with lung cancer screening for high-risk smokers?

Probe: any patients referred, why or why not

1. What are some of the main factors that influence whether or not you might send a patient for lung cancer screening?
2. How might a patient’s social situation impact lung cancer risk and whether or not you offer lung cancer screening?
3. From your perspective, what are the *most important* *barriers* to participation in lung cancer screening for individuals living with low income? Probe: patient, provider, system level barriers.
4. From your perspective, what would be the *most important* *facilitators* to lung cancer screening for individuals living with low income? Probe: patient, provider, system level enablers.
