# Supplementary material for: Perspectives of family physicians towards access to lung cancer screening for individuals living with low income – a qualitative study
Source: BMC Fam Pract. 2021 Jan 7;22:10. doi: 10.1186/s12875-020-01354-z (PMC7791696; doi:10.1186/s12875-020-01354-z)
Supplement: Supplementary file 2 — Additional file 2. [file 12875_2020_1354_MOESM2_ESM.doc]

**Supplemental Table S2: Consolidated criteria for reporting qualitative studies (COREQ) [1]**

| **Item** | **Description** | **Response** | **Reported in section and page number or not applicable N/A** |
| --- | --- | --- | --- |
| **Domain 1: Research team and reflexivity** | | |  |
| *Personal Characteristics* | | |  |
| 1. Interviewer/facilitator | Which author/s conducted the interview or focus group? | AS conducted all of the interviews | Methods, data collection, page 5 |
| 2. Credentials | What were the researcher’s credentials? E.g. PhD, MD | AS- MD, MSc, PhD;  AL – MD, PhD, CCFP;  MV- PhD, MHsc, RN  MAO –PhD  SH – MD, MPH  PS- MBBS, MHSc,CCFP(AM)  GL – MD, MSc, FRCPC  EN - MHSc | Methods, page 4 - 6 |
| 3. Occupation | What was their occupation at the time of the study? | AS – Lead Qualitative Researcher;  AL, SH, PS, GL – Clinician Scientist;  MAO – Qualitative researcher;  MV – Mixed methods researcher;  EN – Federal health care administrator | Methods, page 4 - 6 |
| 4. Gender | Was the researcher male or female? | Female: AS, AL, MV, MAO, EN;  Male: SH, GL, PS | Principal author gender – methods, data collection page 5 |
| 5. Experience and training | What experience or training did the researcher have? | AS, MAO are experienced qualitative researchers  MV is a mixed methods researcher;  AL and GL are clinical epidemiologists;  SH is researcher on homelessness and health  PS is a quantitative researcher in smoking cessation  EN has experience in policy, program development and health services delivery. | Methods, page 4 – 6 |
| *Relationship with participants* | | |  |
| 6. Relationship established | Was a relationship established prior to study commencement? | AS: Apart from one participant, no prior association with participants existed prior to study commencement. | Methods, participant recruitment, page 5 |
| 7. Participant knowledge of the interviewer | What did the participants know about the researcher? e.g. personal goals, reasons for doing the research | Participants received a study information form which explained the purpose of the study. | Methods, participant recruitment, page 4 |
| 8. Interviewer characteristics | What characteristics were reported about the interviewer/facilitator? e.g. Bias, assumptions, reasons and interests in the research topic | AS and AL conduct research on health inequities. This is reported in the manuscript. | Methods, participant recruitment, page 5 |
| **Domain 2: study design** | | |  |
| *Theoretical framework* | | |  |
| 9. Methodological orientation and Theory | What methodological orientation was stated to underpin the study? e.g. grounded theory, discourse analysis, ethnography, phenomenology, content analysis | Qualitative study with theory informed thematic analysis. | Methods, study design, page 4 |
| *Participant selection* | | |  |
| 10. Sampling | How were participants selected? e.g. purposive, convenience, consecutive, snowball | Purposive sampling was used. | Methods, participant recruitment, page 4 |
| 11. Method of approach | How were participants approached? e.g. face-to-face, telephone, mail, email | Participants were approached via email. | Methods, participant recruitment, page 4 |
| 12. Sample size | How many participants were in the study? | 11 participants were included in the study. | Methods, participant recruitment, page 5 |
| 13. Non-participation | How many people refused to participate or dropped out? Reasons? | None of the participants dropped out. 69 potential participants did not respond to our email invitations | Methods, participant recruitment, page 5 |
| *Setting* | | |  |
| 14. Setting of data collection | Where was the data collected? e.g. home, clinic, workplace | Data was collected through telephone interviews and field notes. | Methods, data collection, page 5 |
| 15. Presence of non-participants | Was anyone else present besides the participants and researchers? | Interviews were done via direct telephone | Methods, data collection, page 5 |
| 16. Description of sample | What are the important characteristics of the sample? e.g. demographic data | Sex and percentage of patient population experiencing low income is reported. | Methods, participant recruitment, page 5 |
| *Data collection* | | |  |
| 17. Interview guide | Were questions, prompts, guides provided by the authors? Was it pilot tested? | The interview guide is available from the authors. The guide was pilot tested. | Methods, data collection, page 5 & additional file 1 |
| 18. Repeat interviews | Were repeat interviews carried out? If yes, how many? | Repeat interviews were not carried out. | N/A |
| 19. Audio/visual recording | Did the research use audio or visual recording to collect the data? | Audio-recording was used. | Methods, data collection, page 5 |
| 20. Field notes | Were field notes made during and/or after the interview or focus group? | Field notes were made by AS during the phone interviews | Methods, data collection, page 5 |
| 21. Duration | What was the duration of the interviews or focus group? | Approximately 30- 45 minutes | Methods, data collection, page 5 |
| 22. Data saturation | Was data saturation discussed? | Yes, conceptual saturation is discussed. | Methods, data analysis, page 6 |
| 23. Transcripts returned | Were transcripts returned to participants for comment and/or correction? | No. The transcripts were not returned to participants. We used peer debriefing in place of member checking. | Methods, data analysis, page 6 |
| **Domain 3: analysis and findings** | | |  |
| *Data analysis* | | |  |
| 24. Number of data coders | How many data coders coded the data? | AS and AL coded the data. | Methods, data analysis, page 6 |
| 25. Description of the coding tree | Did authors provide a description of the coding tree? | Coding tree was developed based on conceptual framework of the study | Methods, data analysis, page 6 |
| 26. Derivation of themes | Were themes identified in advance or derived from the data? | Themes were identified from the data. | Methods, data analysis, page 6 |
| 27. Software | What software, if applicable, was used to manage the data? | NVivo Version 12 was used. | Methods, data analysis, page 6 |
| 28. Participant checking | Did participants provide feedback on the findings? | The participants did not provide feedback on the findings. | Methods, data analysis, page 6 |
| *Reporting* | | |  |
| 29. Quotations presented | Were participant quotations presented to illustrate the themes / findings? Was each quotation identified? e.g. participant number | Yes. Each quotation is identified by the type of participant and the setting. | Table 2 |
| 30. Data and findings consistent | Was there consistency between the data presented and the findings? | Consistency between the data and findings. | Table 2 |
| 31. Clarity of major themes | Were major themes clearly presented in the findings? | The major themes are clearly presented. | Table 2 and Figure 1 |
| 32. Clarity of minor themes | Is there a description of diverse cases or discussion of minor themes? | Minor themes are discussed including outlier themes. | Results, page 9, & Figure 1 |

Reference: Tong A, Sainsbury P, Craig J. Consolidated criteria for reporting qualitative research (COREQ): a 32-item checklist for interviews and focus groups International Journal for Quality in Health Care 2007;19:349–357
